# *Streptomyces* sp. MUM256: A Source for Apoptosis Inducing and Cell Cycle-Arresting Bioactive Compounds against Colon Cancer Cells

**DOI:** 10.3390/cancers11111742

**Published:** 2019-11-06

**Authors:** Loh Teng-Hern Tan, Chim-Kei Chan, Kok-Gan Chan, Priyia Pusparajah, Tahir Mehmood Khan, Hooi-Leng Ser, Learn-Han Lee, Bey-Hing Goh

**Affiliations:** 1 Novel Bacteria and Drug Discovery (NBDD) Research Group, Microbiome and Bioresource Research Strength, Jeffrey Cheah School of Medicine and Health Sciences, Monash University Malaysia, Bandar Sunway 47500, Selangor Darul Ehsan, Malaysia; tenghern@gmail.com or loh.teng.hern@monash.edu (L.T.-H.T.); hooileng_ser@y7mail.com (H.-L.S.); 2 Institute of Biomedical and Pharmaceutical Sciences, Guangdong University of Technology, Guangzhou 510006, China; 3 de Duve Institute, Université catholique de Louvain, Avenue Hippocrate 74, 1200 Brussels, Belgium; chimkei@gmail.com; 4 International Genome Centre, Jiangsu University, Zhenjiang 212013, China; 5 Division of Genetics and Molecular Biology, Institute of Biological Sciences, Faculty of Science, University of Malaya, Kuala Lumpur 50603, Malaysia; 6 Medical Health and Translational Research Group, Jeffrey Cheah School of Medicine and Health Sciences, Monash University Malaysia, Bandar Sunway 47500, Malaysia; priyia.pusparajah@monash.edu; 7 Institute of Pharmaceutical Science, University of Veterinary and Animal Science Lahore, Punjab 54000, Pakistan; tahir.khan@uvas.edu.pk; 8 Health and Well-Being Cluster, Global Asia in the 21st Century (GA21) Platform, Monash University Malaysia, Bandar Sunway 47500, Malaysia; 9 Key Laboratory of Microbial Metabolism, Joint International Research Laboratory of Metabolic and Developmental Sciences, School of Life Sciences and Biotechnology, Shanghai Jiao Tong University, Shanghai 200240, China; 10 Biofunctional Molecule Exploratory (BMEX) Research Group, School of Pharmacy, Monash University Malaysia, Bandar Sunway 47500, Selangor Darul Ehsan, Malaysia; 11 College of Pharmaceutical Sciences, Zhejiang University, 866 Yuhangtang Road, Hangzhou 310058, China

**Keywords:** *Streptomyces*, mangrove, anti-proliferative, apoptosis, colon cancer

## Abstract

New and effective anticancer compounds are much needed as the incidence of cancer continues to rise. Microorganisms from a variety of environments are promising sources of new drugs; *Streptomyces* sp. MUM256, which was isolated from mangrove soil in Malaysia as part of our ongoing efforts to study mangrove resources, was shown to produce bioactive metabolites with chemopreventive potential. This present study is a continuation of our previous efforts and aimed to investigate the underlying mechanisms of the ethyl acetate fraction of MUM256 crude extract (MUM256 EA) in inhibiting the proliferation of HCT116 cells. Our data showed that MUM256 EA reduced proliferation of HCT116 cells via induction of cell-cycle arrest. Molecular studies revealed that MUM256 EA regulated the expression level of several important cell-cycle regulatory proteins. The results also demonstrated that MUM256 EA induced apoptosis in HCT116 cells mediated through the intrinsic pathway. Gas chromatography-mass spectrometry (GC-MS) analysis detected several chemical compounds present in MUM256 EA, including cyclic dipeptides which previous literature has reported to demonstrate various pharmacological properties. The cyclic dipeptides were further shown to inhibit HCT116 cells while exerting little to no toxicity on normal colon cells in this study. Taken together, the findings of this project highlight the important role of exploring the mangrove microorganisms as a bioresource which hold tremendous promise for the development of chemopreventive drugs against colorectal cancer.

## 1. Introduction

Colorectal cancer (CRC) constitutes the third most common cancer diagnosed and the fourth leading cause of cancer mortality globally [1]. According to Arnold et al. [2], CRC incidence and mortality is correlated well to human development levels. The study described that the rate of CRC cases is increasing rapidly in many low-income and middle-income countries while stable or decreasing trends are seen in highly developed countries [2]. Treatment of CRC usually involves surgical removal alone when detected early whereas late stage of advanced or metastasized CRC requires radiotherapy or chemotherapy [3]. However, chemotherapy suffers from significant limitations including insufficient selectivity for tumor cells, leading to many adverse side effects [4,5]. Although targeted therapies have been regarded as the most successful treatments of cancer for the past few decades, genetic mutations greatly affect the efficacy of various targeted drug therapies and leading to mutation-driven resistance of targeted therapy [6,7,8]. Thus, there is an urgent need to search for alternative chemotherapeutic/chemoprevention agents which may overcome the limitations of chemotherapy, and natural products are an excellent resource to explore [9].

Natural products have provided the inspiration for the development of alternative agents for the prevention or therapy of cancer, and using natural products to reduce cancer prevalence has received substantial attention [10,11,12,13,14]. The natural products of interest can be found in fruits, vegetables, herbs and medicinal plants [15,16,17,18,19]. Aside from plant-based natural products, microorganisms are also reported to be a useful source of bioactive compounds, including chemotherapeutic agents [20,21,22,23]. For instance, actinomycin was discovered as the first microbial metabolite isolated from the bacterium *Streptomyces antibioticus* in 1940 [24] to be used in cancer therapy. Since then, many more microbial metabolites with antitumor properties were discovered including anthracyclines, bleomycin, mitosanes, mithramycin, pentostatin and calicheamicins [25]. Currently, there is evidence demonstrating that the mangrove derived microbial metabolites could be the next bioresources for potential cancer therapeutic agents [26,27,28,29]. Thus, we explored the potential of *Streptomyces* isolated from Malaysian mangrove soil with a focus on its ability to produce metabolites exhibiting chemopreventive activity.

**Figure 1:**
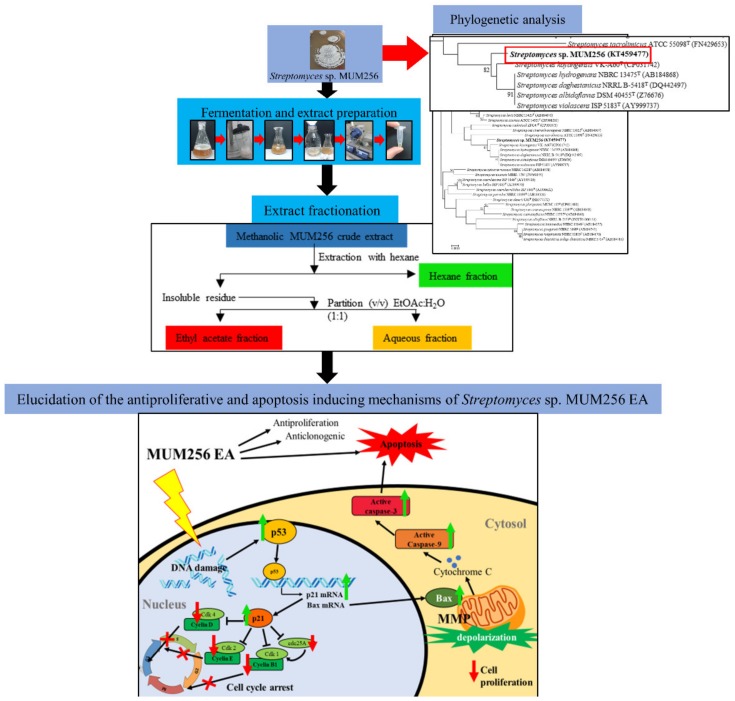
The summarized flow chart of this study. The figure illustrates the fermentation, crude extract extraction, fractionation and elucidated mechanisms of MUM256 EA in cell-cycle arrest and apoptosis induction.

This work represents part of an ongoing project to discover anticancer compounds from mangrove resources, and our screening of the various isolated *Streptomyces* strains led to the discovery of *Streptomyces* sp. MUM256 which possesses the potential to produce active metabolites that induced cell-cycle arrest and apoptosis. In the earlier study [30], we demonstrated that the methanol extract of *Streptomyces* sp. MUM256 exhibited antioxidant and cytotoxic properties. The present study is a continuation of this work aiming to investigate the underlying mechanisms of the cytotoxic and antiproliferative effects of the ethyl acetate fraction of *Streptomyces* sp. MUM256 crude extract (MUM256 EA) against the HCT116 cell line. We demonstrated that the MUM256 EA induced cell-cycle arrest by downregulating several important cell-cycle regulatory proteins and induced apoptosis via interactions with the intrinsic pathway in colon cancer cells (Figure 1). Thus, we believe these results provide new insight into the development of mangrove-derived *Streptomyces* metabolites against CRC.

## 2. Results

### 2.1. Phylogenetic Analysis of Streptomyces sp. MUM256 

**Figure 2:**
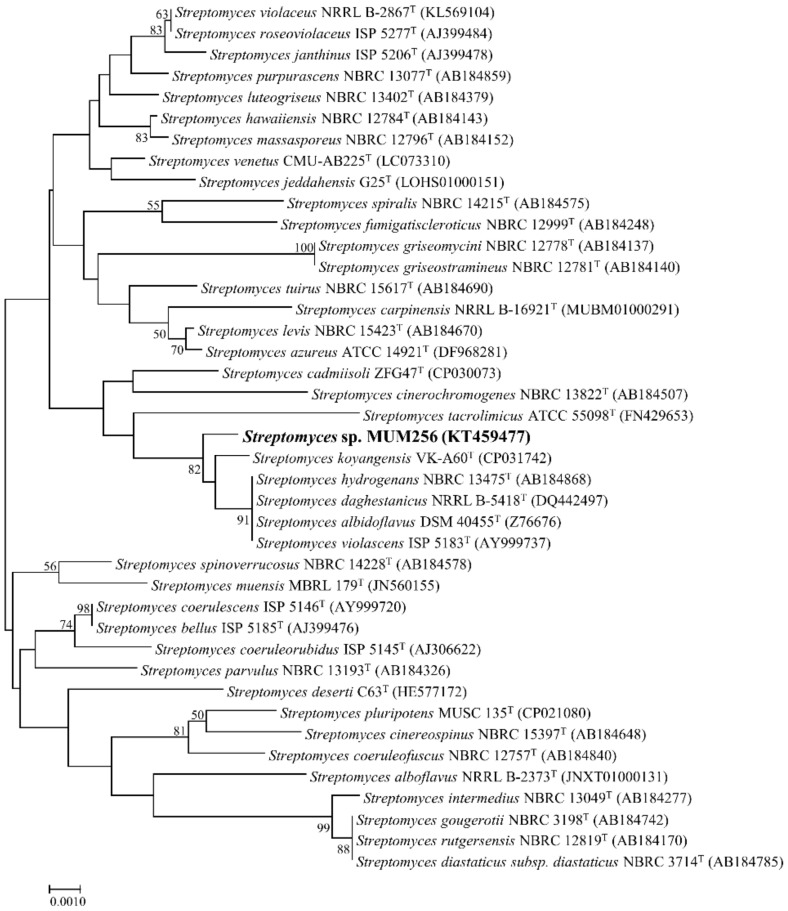
Neighbour-joining phylogenetic tree based on 16S rRNA gene sequence of strain MUM256 (1343bp). The tree illustrates the relationship between strain MUM256 and closely related strains. Numbers at nodes indicate percentages of 1000 bootstrap re-samplings. Bar, 0.001 substitutions per site.

Given that the publicly available database for 16S rRNA gene sequence, such as Ezbiocloud, is regularly updated by adding new bacteria species with validly published names, a new phylogenetic tree was constructed for strain MUM256 based on its 16S rRNA gene sequence (GenBank accession number KT459477) (Figure 2). Based on the blast result of the Ezbiocloud database, the 16S rRNA gene sequence of strain MUM256 demonstrated highest similarity to *S. hydrogenans* NBRC13475^T^ (99.70%), *S. daghestanicus* NRRL B-5418^T^ (99.70%), *S. albidoflavus* DSM40455^T^ (99.70%), *S. violascens* ISP5183^T^ (99.70%) followed by *S. koyangensis* VK-A60^T^ (99.48%). According to Figure 2, the 16S rRNA sequence of strain MUM256 formed a distinct clade with strains *S. koyangensis* VK-A60^T^, *S. hydrogenans* NBRC13475^T^, *S. daghestanicus* NRRL B-5418^T^, *S. albidoflavus* DSM40455^T^ and *S. violascens* ISP5183^T^ at bootstrap value of 82%, showing relatively high confidence level of the association (Figure 2).

### 2.2. To Examine the Cytotoxic Effect of Streptomyces sp. MUM256 Fractions against Colon Cancer Cell HCT116

**Figure 3:**
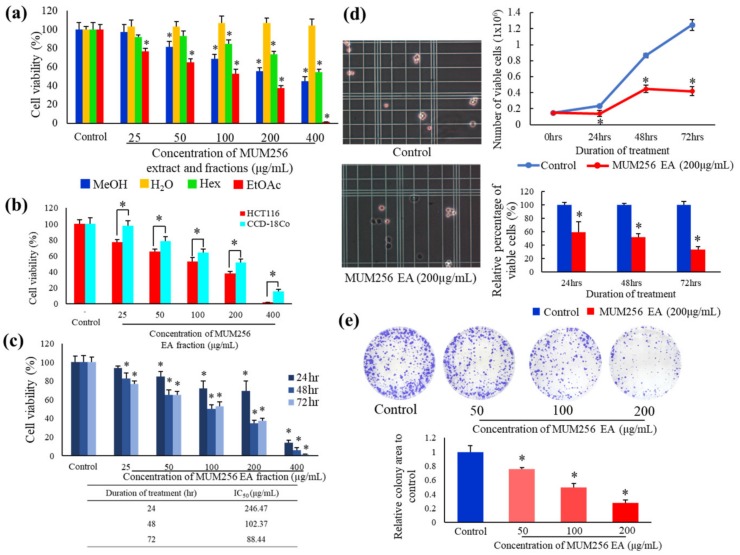
Cytotoxic and antiproliferative properties of MUM256 EA against HCT116 cells. (**a**) Cytotoxic effect of MUM256 crude extract (MeOH: methanol) and 3 different fractions (H_2_O: water; Hex: hexane; EtOAc: ethyl acetate) against HCT116 cells at a series of concentrations after 72 h treatment, (*n* = 4, * *p* < 0.05). (**b**) The toxicity of MUM256 EA towards CCD-18Co as compared to its cytotoxicity against HCT116 after 72 h exposure, (*n* = 4, * *p* < 0.05). (**c**) Cytotoxic effect of MUM256 EA at a series of concentrations against HCT116 cells after 24, 48 and 72 h of exposure, (*n* = 4, * *p* < 0.05). (**d**) Line graph shows the inhibitory effect of MUM256 EA fraction on HCT116 cells. Bar graph illustrates the relative percentage of number of viable cells after exposure to MUM256 EA fraction after normalized to 100% for number of viable cells exposed to vehicle control (0.5% dimethyl sulfoxide (DMSO)) (*n* = 3), * *p* < 0.05 indicates significant difference between the untreated and the treated cells. (**e**) Images of colony formation after 7 days of control and MUM256 EA pre-treated cells in 6 well-plate. Crystal violet staining was used for visualization and quantification of colonies. The area of colonies formed are shown as bar graph with the mean relative mean colony area to control ± standard deviation (SD) (*n* = 3, * *p* < 0.05).

Three different fractions were obtained from the methanolic MUM256 extract after being subjected to sequential fractionation with three types of solvents, namely hexane, ethyl acetate and water. Figure 3a demonstrates the cell viability of HCT116 after exposure to MUM256 extract and the respective fractions for 72 h. The ethyl acetate fraction of MUM256 extract was shown to exhibit the highest cytotoxicity towards HCT116 among the fractions tested, followed by the hexane fraction and the aqueous fraction as the least toxic against HCT116 cells. The toxicity of MUM256 EA was also evaluated on a normal colon cell line CCD-18Co. The MUM256 EA exhibits significantly lesser toxicity towards a normal colon cell (CCD-18Co) at all the concentrations tested in this study (Figure 3b). The IC_50_ of MUM256 EA towards CCD-18Co was measured at 215 μg/mL which is 1.72 higher than its cytotoxicity towards colon cancer cell (HCT116) with IC_50_ of 88.44 μg/mL. This result demonstrates that the MUM256 EA displays a slight preferential cytotoxicity against HCT116 colon cancer cells over a CCD-18Co normal colon cell. 

### 2.3. MUM256 EA Suppresses Cell Viability and Proliferation in HCT116 Cells

To further examine the cytotoxic effects of MUM256 EA using MTT assay, the HCT116 cells were treated with increasing concentrations of MUM256 EA for 24, 48 and 72 h. As shown in Figure 3c, when treated for 24, 48 or 72 h in the concentration range of 25 to 400 μg/mL, MUM256 EA reduced the cell viability of HCT116 cells; MUM256 EA demonstrated cytotoxic effect against HCT116 cells in a significant dose-dependent manner at both 48 and 72 h timepoints. At 400 μg/mL, the cell viability of HCT116 was reduced by MUM256 EA in a time-dependent manner. The concentrations of MUM256 EA required to produce a 50% reduction in cell viability (IC_50_) were determined by a regression analysis; the value of the half maximal inhibitory concentration (IC_50_) decreases according to the time of treatment (IC_50_-24 h: 246.47 μg/mL > IC_50_-48 h: 102.37 μg/mL > IC_50_-72 h: 88.44 μg/mL).

Trypan blue dye exclusion assay showed that the growth of HCT116 cells were reduced upon MUM256 EA treatment as demonstrated by the lower number of viable HCT116 cells in the wells treated with MUM256 EA when compared to the wells with untreated cells (Figure 3d). According to Figure 3d, a significantly lower number of viable cells were detected following exposure to 200 μg/mL MUM256 EA: 59.7%, 51.7% and 33.6% at 24, 48 and 72 h, respectively, when compared to control.

To further evaluate MUM256 EA’s inhibitory effects on cancer cell proliferation, we performed colony formation/clonogenic survival assay on HCT116 cells upon exposure to MUM256 EA treatment, as a test reflecting long-term effect/toxicity [31]. The assay clearly demonstrated that clone formation of HCT116 cells was reduced in a dose-dependent manner (Figure 3e). Treatment with MUM256 EA resulted in a reduction in both numbers of colonies formed as well as the size of the colonies-colony numbers for treated samples were significantly reduced and appeared smaller than those of the control. Therefore, these results confirm that MUM256 EA is cytotoxic and anti-proliferative towards HCT116 cells.

### 2.4. Morphological Changes Induced by MUM256 EA

**Figure 4:**
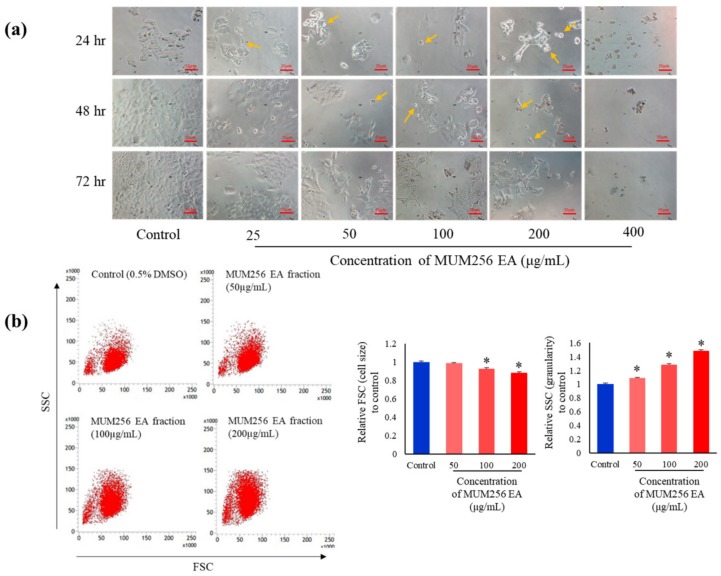
Morphological changes of HCT116 cells after exposure to MUM256 EA treatment. (**a**) Phase contrast microscopic evaluation of HCT116 cells under ×10 magnification. Decreased number of HCT116 cells were observed across the 3 timepoints with increasing concentration of MUM256 EA treatment. Furthermore, arrows indicate the rounded cells due to cytoplasmic shrinkage and detached cells which was increased in number with increasing concentration of MUM256 EA treatment. (**b**) Representative forward scatter/side scatter (FSC/SSC) dot plot of HCT116 cells after exposure to MUM256 EA at different concentrations. The x-axis shows a decreasing FSC in HCT116 cells treated with increasing concentrations of MUM256 EA. The FSC represents the diffracted light and it is related to cell size. The y-axis shows an increasing SSC in HCT116 treated with increasing concentrations of MUM256 EA. The SSC represent the reflected and refracted light and is related to cell granularity and complexity. The bar graphs show the relative median FSC and SSC fluorescence signals of treated cell population as compared to control ± SD (n = 3, * *p* < 0.05).

The morphological examination revealed that the treatment of MUM256 EA visibly altered the cell morphology of the viable HCT116 cells. In Figure 4a, most of the untreated HCT116 cells (control) appeared as normal angular and spindle shapes, but most of the cells lost these features after treatment with MUM256 EA. For example, it was observed that the treatment caused rounding and detachment of HCT116 cells. In addition, reduced number of cells and cell shrinkage with lesser cytoplasm mass were also observed (indicated by arrows) in Figure 4a. These abnormal morphological changes of the cells upon exposure to MUM256 EA has provided some insight on its cytotoxic effect towards the HCT116 cells. 

Furthermore, flow cytometry was also utilized to examine the morphological changes of the cells after treatment with MUM256 EA. The results showed a decrease in forward light scatter (Figure 4b); the median channel fluorescence was lowered in the treated cells with increasing dose of treatment. Meanwhile, the side light scatter was increased in the treated cells significantly with a 1.48-fold increase in the median channel fluorescence compared to the control (Figure 4b). The reduced forward light scatter indicates cell shrinkage, whereas the increased side scatter may be caused by chromatin condensation, nuclear fragmentation and crosslinking of cytoplasmic proteins by activated transglutamine, resulting in the cell becoming more reflective and refractive [32].

### 2.5. Cell Cycle Arrest Effect of MUM256 EA in HCT116 Cells

**Figure 5:**
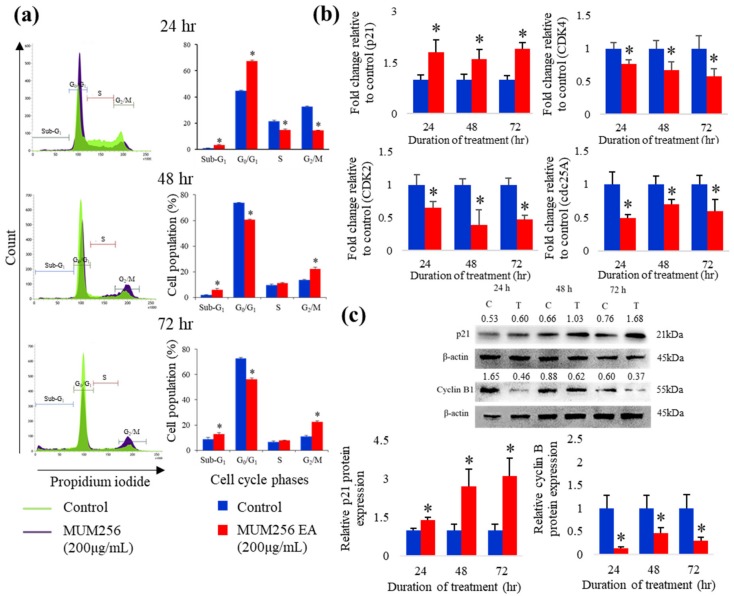
Cell-cycle arrest at G1 and G2 phase induced by MUM256 EA through modulation of cell cycle regulatory proteins. (**a**) Effect of MUM256 EA on cell cycle distribution. HCT116 cells were treated with 200 μg/mL of MUM256 EA for indicated durations (24, 48 and 72 h). Cells were harvested and fixed with ice-cold 70% ethanol overnight. Before flow cytometric analysis, the cells were incubated in phosphate-buffered saline (PBS) containing 25 μg/mL PI, 0.1% Triton-X-100 and 100 μg/mL RNase A for 30 min in the dark at room temperature. The cellular DNA content was determined by flow cytometry. Representative of histograms showing X-axis, DNA contents; y-axis, cell population (%). Each bar represents at least three individual experiments, (* *p* < 0.05). (**b**) Real-time polymerase chain reaction (PCR) analysis of p21, CDK2, CDK4 and cdc25A expressions in HCT116 cells treated with 200μg/mL MUM256 EA. The fold change of the gene was normalized against 18S rRNA expression using the formula 2^−∆∆CT^. Bar graph represents the comparison between treatment and control by *t*-test, * *p* < 0.05 (*n* = 3). (**c**) The levels of p21 and cyclin B1 proteins were determined by Western blotting. HCT116 cells were treated with DMSO and MUM256 EA at 200 μg/mL for indicated durations and the expression of p21 and cyclin B1 were determined by Western blotting with specific antibody. One representative Western blot of three is presented (Figure S1). Each has the expression of β-actin as the internal control. Protein expression was quantified by the densitometry analysis using Image J and normalized against β-actin expression. The values above each band indicate the densitometry ratio of each protein of interest normalized against respective β-actin expression. Bar graph represents the comparison between treatment and control by *t*-test, * *p* < 0.05 (*n* = 3).

In Figure 5a, exposure of HCT116 to 200 μg/mL of MUM256 EA resulted in a statistically significant increase in the percentage of cells in G_0_/G_1_ phase from 44.7% (untreated cells) to 67.4%, with a concomitant decrease in percentage of cells in the G_2_ phase from 32.68% (untreated cells) to 14.31%. However, by 48 h, cells started accumulating in the G_2_ phase from 13.74% to 22.13% and followed by accumulation from 11.14% to 22.39% by 72 h (Figure 5a). These results suggested that MUM256 EA hampers cell-cycle progression by arresting the cells in G_1_ and G_2_/M phase, subsequently lead to inhibition of cell proliferation.

#### Expressions of Cell-Cycle Regulatory Proteins

After treatment with MUM256 EA for 24, 48 and 72 h, both reverse-transcription polymerase chain reaction (RT-PCR) and Western blotting analysis showed the expression levels of p21^Waf1/Cip1^ were significantly elevated as compared to untreated HCT116 cells (Figure 5b,c). As one of the transcriptional targets of p53, the expressions of p21^Waf1/Cip1^ could be regulated by p53 [33,34,35]. Thus, the changes of p53 in response to MUM256 EA in HCT116 was also investigated using intracellular flow cytometry. The p53 protein expression was shown to be increased upon treatment with MUM256 EA for 24 h (Figure 8c). This result supports that p53 activation plays a role in MUM256 EA-induced p21 up-regulation in HCT116 cells.

Besides that, this study also demonstrated that the MUM256 EA treatment downregulated several important cell cycle regulator genes, including CDK2, CDK4 and cdc25A phosphatase (Figure 5b). Western blot analysis also showed that MUM256 EA induced reduction in cyclin B1 protein levels in the HCT116 cells after 24, 48 and 72 h of exposure (Figure 5c).

### 2.6. Nuclear Condensation and DNA Fragmentation

**Figure 6:**
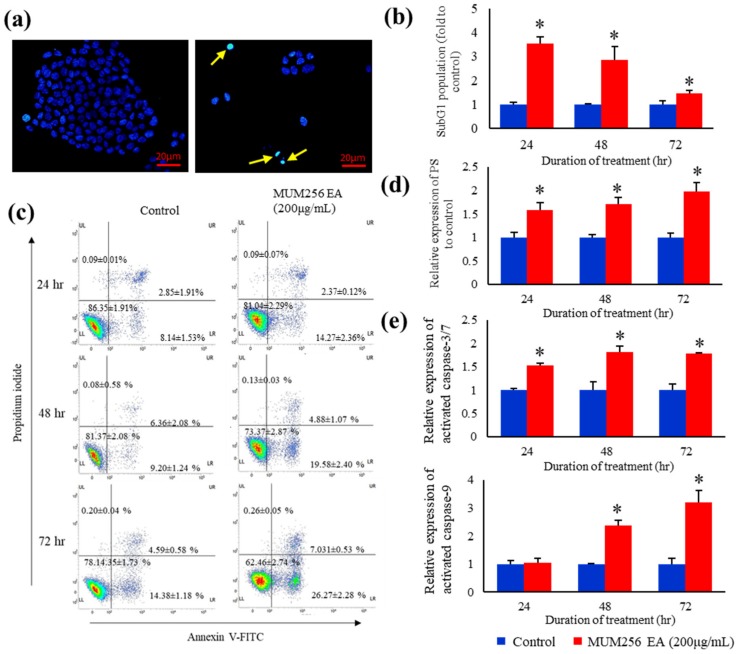
Apoptosis evaluation of MUM256 EA on HCT116 cells. (**a**) Nuclear morphology of HCT116 stained by Hoechst 33342 dye. Comparison of the nuclear morphological features of untreated and treated HCT116 under fluorescence microscope. Arrow indicates the abnormal nuclear morphological features (nuclear shrinkage or condensation shown by the bright-blue fluorescent and nuclear fragmentation) resulted from the cytotoxic effect of MUM256 EA fraction, indicating sign of apoptosis. (**b**) Bar graph represents the mean percentage of subG1 population ± S.D. (*n* = 3, * *p* < 0.05). (**c**) The apoptosis-inducing effect of MUM256 EA fraction was examined by flow cytometric analysis using double staining of Annexin V-FITC and PI on HCT116 cells for 24, 48 and 72 h. Quantification of early apoptosis (LR) (Annexin V positive/PI negative) and late apoptosis (UR) (Annexin V positive/PI positive) are shown by the representative four quadrants plot. The bar graph represents the mean percentage of population ± S.D., (*n* = 3, * *p* < 0.05). (**d**) Both the early apoptotic portion and the late apoptotic portion were included as the total apoptotic cells upon treatment for 24, 48 and 72 h. The bar graph represents the mean percentage of apoptotic cells ± S.D, (*n* = 3, * *p* < 0.05). (**e**) Flow cytometry analysis of caspase-3/7 and 9 activities in HCT116 cells using a non-cytotoxic fluorescent labelled inhibitors of caspase-3/7 and 9. HCT116 cells were treated with 200 µg/mL MUM256 EA fraction for various times as indicated, or with DMSO as a control, then stained with FAM-DEVD-FMK or FAM-LEHD-FMK which recognizes and binds to active caspase-3/7 or 9, respectively, before analysed by flow cytometry (BD FacVerse). The bar graph represents the median of fluorescence intensity (MFI) of active caspase-3/7 or 9 straining in treated cells normalized to control ± SD. (*n* = 3, * *p* < 0.05).

Differences were observed in the nuclei of treated and untreated HCT116 after staining with Hoechst 33258 (Figure 6a). The Hoechst 33258 dye stains morphologically normal nuclei dimly blue, whereas treated cells demonstrate smaller nuclei with brilliant blue staining due to nuclear condensation (yellow arrows). This result demonstrated that MUM256 EA induces morphological changes with characteristics of apoptotic cell death. This result is also consistent with the detection of a significantly increased percentage of subG1 cell populations as an index of the apoptotic DNA fragmentation.

MUM256 EA treatment increased the percentage of cells in the subG_1_ phase after treatment for 24, 48 and 72 h, respectively, as compared to control (Figure 6b). Intranucleosomal DNA fragmentation is a major hallmark of apoptosis [36,37]. PI is a fluorescent dye that binds stoichiometrically to nucleic acids [38]; and as fluorescence emission is proportional to DNA content of the cells, it allows the analysis of a cell population’s replication state. Hence, cells undergoing apoptosis can be identified from the DNA content histograms as those with lower fluorescent signal due to the low molecular weight of DNA fragments present in these cells compared to the G1 cells [37]. These data suggest that the reduced cell proliferation by MUM256 EA could be contributed by the induction of apoptosis.

### 2.7. Exposure of Phosphatidylserine 

In this study, additional evidence for the occurrence of apoptosis was obtained by double staining of the cells with propidium iodide (PI) and Annexin V-FITC. Annexin V is a protein that binds with high affinity to phosphatidylserine (PS), which is translocated from the inner to the outer membrane leaflet during early apoptosis. Meanwhile, the healthy cells have phosphatidylserines on the inner leaflet of the plasma membrane. This technique is then combined with DNA binding dye (propidium iodide) which is impenetrable to intact membrane, hence allowing for the determination of the apoptotic state of a cell. As shown in Figure 6c, the percentage of the early apoptotic cells (PS positive and PI negative cells) increased in response to treatment with MUM256 EA for 24, 48 and 72 h. Overall, the percentage of apoptotic cells (PS positive cells) were increased in HCT116 cells treated with MUM256 EA for 24, 48 and 72 h (Figure 6d). 

### 2.8. Caspase Activation

**Figure 7:**
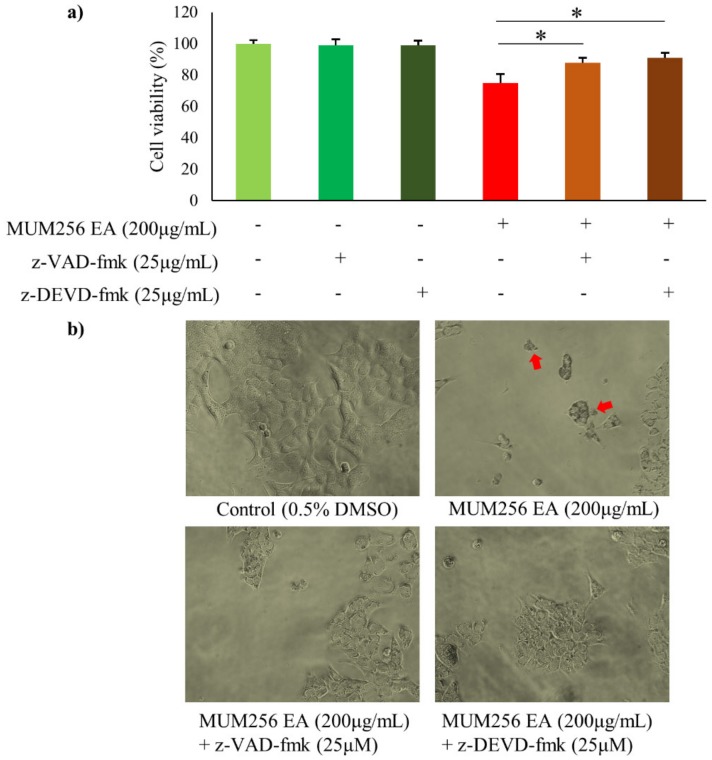
Effect of caspase inhibitors on MUM256 EA induced apoptosis in HCT116 cells. (**a**) Cells were pretreated with either z-VAD-fmk (25 μg/mL) or z-DEVD-fmk (25 μg/mL) for 1 h, then treated as described (Control—0.5% DMSO, with or without—200 μg/mL MUM256 EA) for 24 h. Cell viability was measured by MTT (3-(4,5-dimethylthiazol-2-yl)-2,5-diphenyltetrazolium bromide) assay, (n = 4, * *p* < 0.05). (**b**) Morphological changes of HCT116 cells upon indicated treatments were observed by phase-contrast microscopy (magnification ×20). Arrows indicate the treated cells with apoptotic morphology.

To investigate the effect of MUM256 EA in activation of caspases, flow cytometric analysis was performed using non-cytotoxic fluorescent labelled inhibitors, FAM-DEVD-FMK and FAM-LEHD-FMK that bind irreversibly to cysteine of the active center of caspase-3/7 or caspase-9 in the cells, respectively, hence allowing accurate quantification of the caspase activity in the cells. The results showed that the expression of active caspase-3/7 in HCT116 cells were significantly increased upon treatment with MUM256 EA for 24, 48 and 72 h as demonstrated by the increased median fluorescence intensity (MFI) (Figure 6e). Similarly, the flow-cytometric analysis demonstrated that MUM256 EA induced an increasing trend of caspase-9 activation in HCT116 cells (Figure 6e). Thus, MUM256 EA-induced cell death was accompanied by an increase in caspase activities, which then stimulated the molecular cascade of apoptosis. To further validate the apoptosis inducing effect of MUM256 EA, pretreatment of cells with either z-VAD-fmk, a broad-spectrum caspase inhibitor, or z-DEVD-fmk, a caspase-3 inhibitor, diminished the cell death induced by MUM256 EA (Figure 7). These data suggest that the MUM256 EA-induced apoptosis involves a caspase-dependent pathway in HCT116 cells.

### 2.9. Mitochondrial Membrane Potential (MMP)

**Figure 8:**
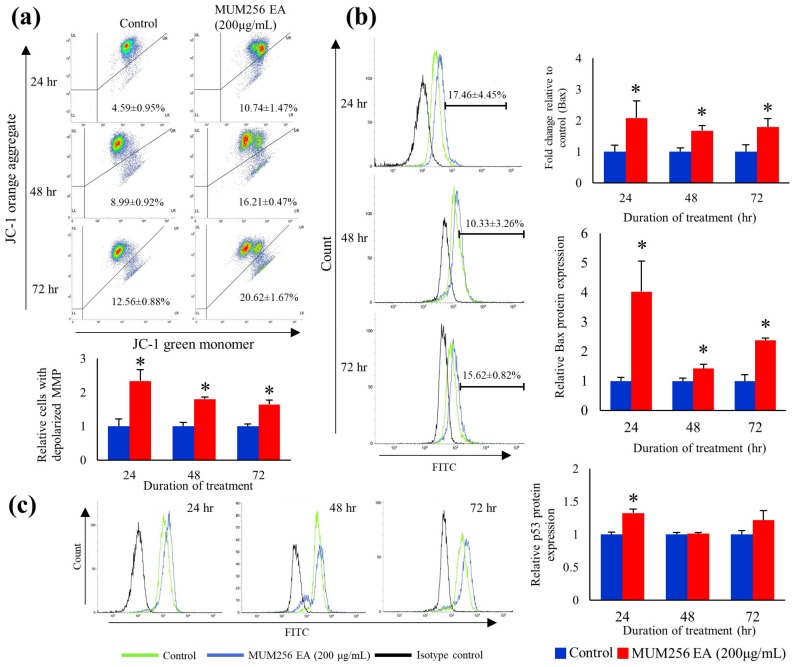
Effect of MUM256 EA on the mitochondrial membrane potential, proapoptotic protein (Bax) and p53 status in HCT116 cells. (**a**) HCT116 cells were treated with MUM256 EA for indicated durations and then were stained with JC-1 dye to detect the change of MMP by flow cytometry. Dot plots are representative of three independent experiments. Bar graph shows the ratio of JC-1 orange/green normalized to control, decrease in the ratio indicating MMP depolarization upon treatment, * *p* < 0.05 (*n* = 3). (**b**) Real-time PCR analysis of Bax expression in HCT116 cells treated with 200 μg/mL MUM256 EA fraction. The fold change of the gene was normalized against porphobilinogen deaminase (PBGD) expression using the formula 2^−∆∆CT^. Flow cytometry analysis of Bax protein expression in HCT116 cells using intracellular immunofluorescence staining method. Representative overlay of histograms showing Bax-associated fluorescence intensity after exposure to MUM256 EA fraction at 200 µg/mL for 24, 48 and 72 h. Bar graph represents the relative expression levels of Bax protein in treated HCT116 cells compared to control. Values are mean percentage of cells with expressed Bax ± SD. of three independent experiments. Asterisks indicate a significant difference compared to control (* *p* < 0.05). (**c**) Flow cytometry analysis of p53 protein expression in HCT116 cells using intracellular immunofluorescence staining method. Representative overlay of histograms showing p53-associated fluorescence intensity after exposure to MUM256 EA fraction at 200 µg/mL for 24, 48 and 72 h. Relative expression levels of p53 protein in HCT116 cells. Values are median fluorescence intensity ± SD of three independent experiments. Asterisks indicate a significant difference compared to control (* *p* < 0.05).

To determine whether the caspase-dependent apoptosis in HCT116 cells was mediated through the mitochondrial apoptotic pathway in response to MUM256 EA treatment, the effect of MUM256 EA on the mitochondrial membrane permeability was investigated. JC-1 dye was used to assess the mitochondrial transmembrane potential of HCT116 cells in response to the treatment with MUM256 EA. JC-1 dye exhibits variable characteristics when bound to the membrane of apoptotic cells versus non-apoptotic cells. In non-apoptotic cells, JC-1 dye appears in aggregate form, emitting orange fluorescence at 590 nm when bound on polarized mitochondrial membrane with high membrane potential; but exists in monomeric form that emits green fluorescence at 527 nm in cells with disrupted mitochondrial potential [39]. The results of flow cytometry showed that treatment of HCT116 cells with MUM256 EA resulted in the increase of green-fluorescence-positive cells as well as the decrease in the JC-1 orange/green ratio in treated cells, suggesting depolarization of MMP (Figure 8a). This result suggested that the MUM256 EA induced cell apoptosis by causing the collapse of mitochondrial transmembrane potential in HCT116 cells.

### 2.10. Bax Pro-Apoptotic Protein Expressions 

Given that mitochondrial transmembrane permeabilization represents a process of point-of-no-return, it is highly regulated, largely by members of the BCL-2 protein family [40]. Thus, we investigated the effect of MUM256 EA on the expression levels of Bax—which is a member of the BCL-2 family—in HCT116 cells. As shown in Figure 8b, RT-PCR analysis showed that treatment of HCT116 cells with MUM256 EA led to significantly increased expression of Bax at 24, 48 and 72 h. In line with the RNA expression result, the intracellular flow cytometry analysis showed the level of proapoptotic protein Bax in HCT116 cells was increased significantly upon treatment with MUM256 EA for 24, 48 and 72 h. This result suggested that the overall increase in Bax expressions could be the contributor to the mitochondrial mediated apoptosis induced by MUM256 EA in HCT116 cells.

### 2.11. Detection of Bioactive Constituents in Streptomyces sp. MUM256 EA Extract Using Gas Chromatography-Mass Spectrometry

**Figure 9:**
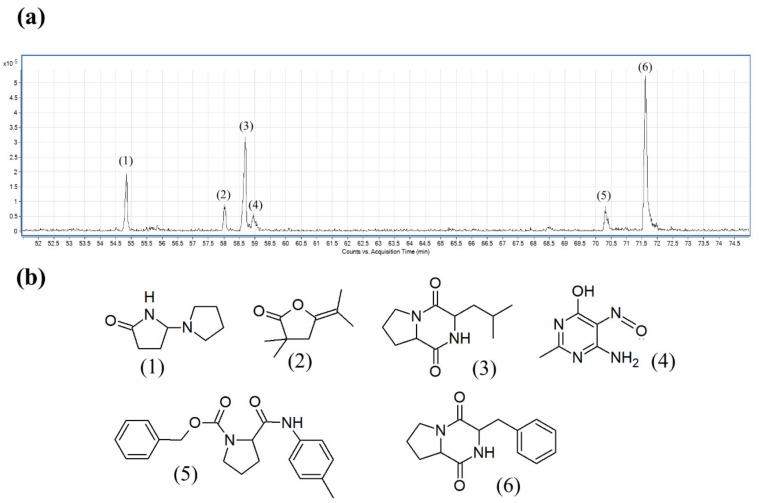
Gas chromatography-mass spectrometry (GC-MS) analysis of MUM256 EA. (**a**) The GC chromatogram of MUM256 EA. (**b**) The chemical structures of the detected constituents present in MUM256 EA. The identification of the chemical compounds was performed by comparing their mass spectra to standard mass spectra available in the database of NIST 05 Spectral library.

To further elucidate the potential chemical compounds that may have contributed to the antioxidant and cytotoxic properties, gas chromatography-mass spectrometry (GC-MS) was used to detect the chemical compounds present in MUM256 EA. GC-MS has been widely used as the analytical tool for molecular detection and identification in drug discovery. Numerous studies also utilized GC-MS to profile the bioactive compounds present in the secondary metabolites of *Streptomyces* bacteria [41,42,43]. The results of GC-MS analysis revealed that MUM256 EA contains several groups of chemical compounds, including the pyrrole, pyrazine and cyclic dipeptides. The identification of the chemical compounds was performed by comparing their mass spectra to standard mass spectra available in the database of NIST 05 Spectral library. Table 1 tabulates the retention time, molecular weight and molecular formula of the chemical compounds. Figure 9a,b depicts the GC chromatogram and chemical structures detected in MUM256 EA, respectively.

### 2.12. The Cytotoxic Effects of the Two Main Bioactive Constituents Detected by Gas Chromatography-Mass Spectrometry (GC-MS) against HCT116 and CCD-18Co Cells

**Figure 10:**
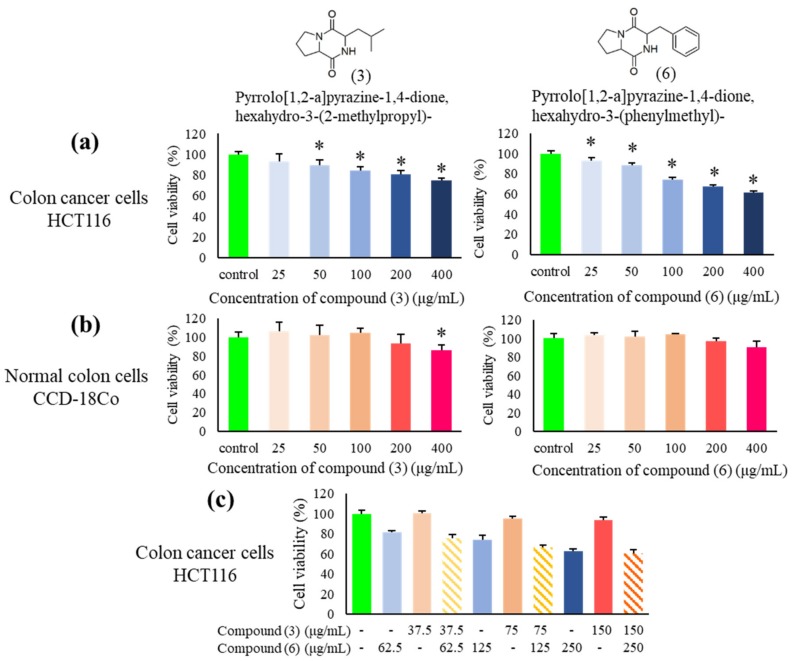
Cytotoxic effects of the two main Compounds **(3)** and **(6)** on HCT116 and CCD-18Co cells. (**a**) Cytotoxic effect of pyrrolo [1,2-a] pyrazine-1,4-dione, hexahydro-3-(2-methylpropyl)- (**3**) and pyrrolo [1,2-a] pyrazine-1,4-dione, hexahydro-3-(phenylmethyl)- (**6**) against HCT116 cells at a series of concentrations after 72 h treatment, (*n* = 5, * *p* < 0.05). (**b**) The toxicity of pyrrolo [1,2-a] pyrazine-1,4-dione, hexahydro-3-(2-methylpropyl)- (**3**) and pyrrolo [1,2-a]pyrazine-1,4-dione, hexahydro-3-(phenylmethyl)- (**6**) towards CCD-18Co after 72 h exposure, (*n* = 5, * *p* < 0.05). (**c**) The combined effect of Compounds (**3**) and (**6**) on HCT116 at respective concentrations after 72 h treatment, (*n* = 5, * *p* < 0.5).

To examine the cytotoxic properties of the chemical compounds in MUM256 EA, pure Compounds (**3**) and (**6**) were obtained and tested against HCT116 colon cancer cells and the CCD-18Co normal colon cells. Based on the results, Compound (**6**) showed stronger cytotoxic effect against HCT116 cells as compared to Compound (**3**) after 72 h exposure (Figure 10a). Interestingly, both compounds were not toxic towards the CCD-18Co cells with no significant reduction in cell viability at all the concentrations tested after 72 h of exposure (Figure 10b), except Compound (**3**) reduced the cell viability of CCD-18Co to 86% at 400 μg/mL. Both compounds also showed cytotoxic effect against another colon cancer (HT-29 cells) (Figure S2). To investigate potential combination effects between Compounds (**3**) and (**6**), HCT116 cells were treated with Compounds (**3**) and (**6**) at a ratio of 3:5 based on peak height shown in the GC-MS total ion chromatogram. However, the results did not indicate any significant combined effect of both compounds against HCT116 (Figure 10c).

## 3. Discussion

*Actinobacteria*, including the genus *Streptomyces*, from unexplored ecosystems, such as mangrove environments, represent a prolific source of bioactive compounds which can contribute immensely to humans in various biotechnological and pharmaceutical applications [44,45,46,47,48]. The capability of these mangrove-derived *Streptomyces* to produce unique secondary metabolites with interesting bioactivities has been linked to the highly dynamic mangrove ecosystem that exerts significant influence on bacterial metabolic and physiological adaptations [26,49]. In the present study, *Streptomyces* sp. MUM256 was demonstrated to produce bioactive compounds—present in the ethyl acetate fraction of crude extract—which exhibited promising antiproliferative activity against colon cancer cells.

In this study, MUM256 EA demonstrated the strongest cytotoxicity effects on HCT116 cells as examined by the 3-(4,5-dimethylthiazol-2-yl)-2,5-diphenyl tetrazolium bromide (MTT) assay which measures the mitochondrial activity in viable cells [50]. Based on the morphological changes assessed by phase-contrast microscopy and flow cytometry, MUM256 EA is suggested to exert its cytotoxic effect by causing the reduced viability of HCT116 through mechanisms associated to apoptotic cell death. This is because among the predominant features of apoptotic cells were their diminished size, condensation of chromatin, segregation of nucleoli, nuclear fragmentation and formation of apoptotic bodies [51,52], features which we observed in treated cells. However, the decreased forward light-scatter signal is not a specific marker of apoptosis since mechanically broken and fragmented cells, isolated cell nuclei, and necrotic cells also have reduced light-scatter properties. Therefore, a more specific assay should be accompanied with these observations to determine the mode of cell death.

Carcinogenesis is a result of cell-cycle disorganization, leading to uncontrolled cellular proliferation [53]. Regulatory proteins like the cyclins, cyclin-dependent kinases (CDK) and their substrate proteins, CDK inhibitors and tumor-suppressor genes are major proteins that control the cell-cycle progression [54]. In tumors, these cell cycle regulators are altered, resulting in failure to control the correct entry and progression through the cell cycles; thus agents that suppress proliferation of cancer cells by regulating these cell cycle regulators have therapeutic value [55]. In the present study, we demonstrated that MUM256 EA reduced the proliferation of colon cancer cells by arresting cells at G1 and G2 phases through the modulation of cell-cycle regulatory proteins including p21, cyclin B1, CDK2, CDK4 and cdc25A phosphatase.

CDK inhibitory protein, p21, is an essential signaling molecule in regulating the cell-cycle progression by interacting directly with cyclin/CDK complexes. It inhibits CDK activities, which in turn results in decreased cell proliferation. Hence, the expression of CDK inhibitory protein p21 has been exploited in the development of chemotherapeutic drugs that disrupt carcinogenesis by impeding the cell cycle in cancer cells [56]. Consistent with this notion, the increased p21 expression level in response to MUM256 EA treatment was accompanied by the decreased expression levels of CDK2 and CDK4 in HCT116 cells. CDK4 associates with cyclin D to regulate the cell-cycle progression in the G1 phase; the cyclin D/CDK4 senses cell mitogenic signals and prompts the cells to initiate DNA synthesis [55,57]. Similarly, CDK2 associates with cyclins E/A to form complexes which activate the downstream targets, including Rb and E2F transcription factor, hence facilitating the transition of G1 to S phase and DNA synthesis [58]. Thus, the accumulation of HCT116 cells in G1 phase in response to MUM256 EA could be due to the downregulation of CDK2 and CDK4 which are crucial in controlling G1/S transition and G1 progression.

The cdc25A is found to be involved in both early G1/S and late G2/M transitions. In the regulation of S phase entry, it activates CDK2 through its dephosphorylating action [59]. Meanwhile, overexpression of cdc25A was also found to mediate mitosis through the regulation of G2/M transition by activating the cyclin B1/CDK1 complexes [60,61]. Thus, we examined the expression levels of cdc25A and cyclin B1 in HCT116 cells upon treatment with MUM256 EA. The expression of cdc25A phosphatase was reduced in HCT116 cells upon treatment with MUM256 EA. Furthermore, a previous study demonstrated that p21 plays an important role in mediating the degradation of cyclin B1 in response to DNA damage and is necessary for the maintenance of G2 cell-cycle arrest in HCT116 cells [62]. Thus, the upregulation of p21 and downregulation of cdc25A in response to MUM256 EA may be involved in the downregulation of cyclin B1 which lead to G2/M cell-cycle arrest in HCT116 cells.

Dysregulation of the apoptotic mechanism has been associated with carcinogenesis. Therefore, any defects along the apoptotic pathway can lead to unchecked cell proliferation, development and progression of cancer and cancer resistance to drug therapies. Thus, apoptosis induction in cancer cells by modulating the pro-apoptotic proteins involved in the apoptotic pathways is an effective strategy for cancer treatment. We demonstrated that MUM256 EA induced cell death in HCT116 via apoptosis as shown by the increased number of Annexin-V positive cells. Furthermore, the use of fluorescent labelled inhibitors, FAM-DEVD-FMK and FAM-LEHD-FMK and the use of caspase inhibitors (benzoylcarbonyl-valyl-alanyl-aspartyl-[O-methyl]-fluoromethylketone (z-VAD-fmk) and benzoylcarbonyl-aspartyl-glutamyl-valyl-aspartyl-fluoromethylketone (z-DEVD-fmk)) further confirmed the role of caspases in MUM256 EA-induced apoptosis. Activated initiator caspases cleave and activate effector caspases, which are involved in cleavage of multiple cellular protein substrates, resulting in triggering of apoptosis [40].

Our data showed that MUM256 EA treatment resulted in increased HCT116 cells with depolarised MMP as compared to the untreated of HCT116 cells. The loss of mitochondrial membrane potential is one of the hallmarks of apoptosis [63]. This result suggests that MUM256 EA-induced cell apoptosis is mediated by mitochondrial-dependent pathways in HCT116 cells. Nevertheless, the occurrence of mitochondrial depolarization also could be a consequence of apoptosis induced by MUM256 EA.

The apoptotic mitochondrial events are tightly regulated by the members of Bcl-2 family of proteins [64]. As a proapoptotic protein, the active Bax functions to permeabilise the mitochondrial outer membrane through the formation of oligomers which induce pore formation at the mitochondrial outer membrane [65]. It is also well established that Bax accumulation causes the release of cytochrome c from mitochondria, leading to the activation of caspase cascade [66,67]. The present findings indicate that MUM256 EA treatment elevated both RNA and protein expression of Bax in HCT116 cells which confirmed the involvement of intrinsic pathway of apoptosis in HCT116 cells.

Literature shows that p53 mediated G_1_ and G_2_ cell cycle arrest could also be accomplished through transcriptional activation of its downstream targets p21, GADD45α, 14-3-3ε [68]. Our results also support this notion that p53 activation may be involved in the cell cycle arrest in HCT116 cells mediated by MUM256 EA-induced p21 up-regulation. Furthermore, p53 plays an important role in apoptosis via induction of pro-apoptotic Bax protein in response to cellular stress responses. Thus, the increased p53 protein expression suggests that p53 activation may also play an important in the MUM256 EA-induced apoptosis in HCT116 cells. Overall, these data suggest that MUM256 EA triggers sequential activation of p53/Bax/caspase-9/caspase-3/7 cascade to induce the mitochondrial-mediated apoptotic cell death program in HCT116 cells.

According to GC-MS analysis, several groups of chemical compounds such as pyrrole, pyrazine and cyclic dipeptides were detected in the MUM256 EA. The chemical compounds detected in this study also have been evidenced previously in the microbial fermentation broth/extracts, including those isolated from Actinobacteria and the genus *Streptomyces*. For example, 5-pyrrolidino-2-pyrrolidone (**1**) [43], pyrrolo [1,2-a] pyrazine-1,4-dione, hexahydro-3-(2-methylpropyl)- (**3**) [69,70], pyrrolo [1,2-a] pyrazine-1,4-dione, hexahydro-3-(phenylmethyl)- (**6**) [70].

Both pyrrolo [1,2-a] pyrazine-1,4-dione, hexahydro-3-(2-methylpropyl)- (**3**) and pyrrolo [1,2-a] pyrazine-1,4-dione, hexahydro-3-(phenylmethyl)- (**6**) were detected and they belong to the chemical group of proline-containing cyclic dipeptide or 2,5-diketopiperazines (DKP). They appear to be the two major constituents present in MUM256 EA (Figure 9a). DKP consists of a group of the simplest peptide derivatives that are found ubiquitously in nature [71]. Numerous studies have also pointed out the detection of these peptides in the fermentation culture of microorganisms [72,73,74]. Previous studies also reported that these cyclic dipeptide compounds possess antioxidant and cytotoxic properties [75,76,77,78]. A number of previous studies also elucidated the underlying mechanism of action of pyrrolo [1,2a] pyrazine-1,4-dione, hexahydro-3-(phenylmethyl)- (**6**) in killing cancer cells [79,80,81]. To sum up the results of GC-MS analysis, several chemical compounds were detected which have been noted to have pharmacological properties by previous studies, suggesting that the anti-colon cancer properties of MUM256 EA could be contributed by these chemical compounds. In this study, both pyrrolo [1,2-a] pyrazine-1,4-dione, hexahydro-3-(2-methylpropyl)- (**3**) and pyrrolo [1,2a] pyrazine-1,4-dione, hexahydro-3-(phenylmethyl)- (**6**) were demonstrated to inhibit the growth of HCT116 cells, indicating that these compounds may have been responsible for the anti-colon cancer properties of MUM256 EA. However, the efficacy of these compounds and their combined effect was not as strong as MUM256 EA against HCT116 colon cells, and this may be due to possible interactions between Compounds (**1**), (**2**), (**4**) and (**5**) in MUM256 EA with the cancer cells. Thus, future study should further identify and validate the potential lead compound as well as elucidate the mechanisms underlying the cell cycle arrest and apoptosis induction. Nonetheless, this study has shed light on the *Streptomyces* sp. strain MUM256 as a promising source for bioactive compounds with anti-colon cancer activities.

## 4. Materials and Methods

### 4.1. Phylogenetic Analysis of Strain MUM256

The 16S rRNA gene of strain MUM256 (GenBank accession number KT459477) was subjected to multiple alignment analysis using CLUSTAL-X software with representative sequences of related type strains in genus *Streptomyces* [82]. The reference sequences were obtained from the GenBank/European Molecular Biology Laboratory (EMBL)/DNA Data Bank of Japan (DDBJ) databases on 20th June 2019. The phylogenetic tree was reconstructed according to neighbor-joining algorithm with the use of MEGA version 6.0 software. Kimura’s two-parameter model was selected for computing the evolutionary distances of neighbor-joining algorithm [83]. Bootstrap analysis based on 1000 resamplings method of Felsentein was performed to evaluate the topology of the constructed phylogenetic tree [84]. The sequence similarities were calculated based on Ezbiocloud server (http://www.ezbiocloud.net/) [85].

### 4.2. Fermentation and Preparation of Extracts for Bioactivities Screening

A 14-day culture of *Streptomyces* sp. MUM256 in TSB was used as the inoculum for fermentation process using Han’s fermentation media 1 (HFM1) as the fermentative medium [49,86]. Fermentation was carried out in an Erlenmeyer flask containing 200 mL of HFM1 for 7–10 days at 28 °C with shaking speed at 200 rpm. The resulting HFM1 medium was subjected to centrifugation at 12,000× *g* for 15 min. The supernatant was filtered and freeze-dried before proceeding to extraction with methanol. After 72 h of extraction with methanol, the supernatant was filtered and collected. The residue was re-extracted twice under the same conditions. A rotary vacuum evaporator was used to remove the methanol from the crude extract at 40 °C, and DMSO was used to dissolve the crude extract prior to assay.

### 4.3. Fractionation of Methanolic Crude Extract of Streptomyces sp. MUM256

The MUM256 crude extract was subjected to sequential fractionation using selected solvents namely hexane, ethyl acetate and water. The solid methanolic MUM256 extract was suspended and macerated with hexane solvent. The hexane fraction was obtained after filtration through filter paper. The residue was partitioned between equal volumes of ethyl acetate and distilled water. Both the hexane and ethyl acetate fractions were concentrated using a rotary evaporator while the aqueous fraction was subjected to freeze drying. The dried fractions were suspended in DMSO before further analysis.

### 4.4. Cell Culture

Both human colon cancer (HCT116, HT-29) and normal colon (CCD-18Co) cell lines were purchased from American Type Culture Collection (ATCC, Virginia, VA, USA). The cells were cultured in RPMI (Roswell Park Memorial Institute)-1640 (Gibco, Life Technologies, Gaithersburg, MD, USA) supplemented with 10% fetal bovine serum and 1× antibiotic-antimycotic (Gibco) at 37 °C in a humidified incubator containing 5% CO_2_ and 95% air. The cultures were viewed using an inverted microscope to assess the degree of confluency and to ensure they were free from bacterial and fungal contamination.

### 4.5. Cell Viability Test Using MTT (3-(4,5-Dimethylthiazol-2-yl)-2,5-Diphenyltetrazolium Bromide) Assay

The cytotoxic effect of the extract/fractions on HCT116, HT-29 and CCD-18Co cell lines were determined using 3-(4,5-dimethylthiazol-2-yl)-2,5-diphenyltetrazolium bromide (MTT) assay [87]. The cells (5 × 10^3^ cells/well) were seeded into 96-well plates and left to adhere overnight in the incubator. *Streptomyces* extract/fraction was added into each well in a series of concentrations (25 to 400 µg/mL) in the presence or absence of apoptosis inhibitors (z-VAD-fmk or z-DEVD-fmk). Pyrrolo [1,2-a] pyrazine-1,4-dione, hexahydro-3-(2-methylpropyl)- (Henan Tianfu Chemical Co., Ltd., Zhengzhou, China) and pyrrolo [1,2-a] pyrazine-1,4-dione, hexahydro-3-(phenylmethyl)- (Chemieliva Pharmaceutical Co., Ltd., Chongqing, China) were added into each well in a series of concentrations. DMSO (0.5% (v/v)) was used as the solvent and the negative control in all the experiments. The treatment of cells was conducted according to the indicated durations. MTT assay was performed with the addition of 20 µL of 5 mg/mL of MTT (Sigma, Saint Louis, MO, USA) into each well and incubated at 37 °C in a humid atmosphere with 5% CO_2_, 95% air for 4 h. The formazan crystals were dissolved in DMSO (100 µL) after the removal of the medium. The absorbance of dissolved formazan solution was measured spectrophotometrically at 570 nm (with 650 nm as reference wavelength) using a microplate reader.

### 4.6. Trypan Blue Exclusion Assay

Trypan blue was used to stain and differentiate live and dead cells. Under phase contrast microscopy, the live cells appear colorless and bright while the dead cells stained blue after exposure to trypan blue. The live cells were counted using a haemocytometer.

### 4.7. Clonogenic Survival Assay

HCT116 cells were seeded in 6-well plates at 1500 cells/well, and the indicated concentrations of MUM256 EA were added the following day. After 24 h of treatment, the media containing the MUM256 EA was aspirated and rinsed with phosphate-buffered saline (PBS). Fresh culture media was added, and the cells were left for 1 week to form colonies [88]. The culture media was replaced with fresh culture media every 2 days. Colonies were fixed in ice-cold methanol and subsequently stained with 0.01% crystal violet in dH_2_O for 10 min. Excess stain was rinsed twice with dH_2_O and allowed to air dry. Images of each well were taken, and the colony area was quantitated using the ‘colony area’ plugin of ImageJ software [89].

### 4.8. Phase Contrast Microscopy

The effect of MUM256 EA on the HCT116 cells was examined morphologically with an inverted light microscope.

### 4.9. Flow Cytometry

Flow cytometric analysis was performed on a BD FACSVerse^TM^ flow cytometer (BD Bioscience, San Jose, CA, USA). For phenotypic characterization of cells, the cells were harvested and washed twice with PBS after treatment. The aliquots of cell suspension were analysed according to cell size (as defined by FSC value) and their granularity (as defined by SSC value) using the FSC/SSC dot plot diagram. A total of 10,000 events were acquired for each sample and presented on the FSC/SSC dot plot.

### 4.10. Cell-Cycle Analysis Using Propidium Iodide (PI)

DNA content and cell cycle distribution were evaluated using propidium iodide (PI) staining [90]. After exposure to MUM256 EA for a designated period, the cells were harvested and washed with PBS before being fixed with 70% ice-cold ethanol at −20 °C overnight. Fixed cells were washed twice and stained in a buffer containing 25 μg/mL PI, 0.1% Triton-X-100 and 100 μg/mL RNase A for 30 min in a dark at room temperature. The PI-stained cells were acquired up to 10,000 events and analysed using BD FACSVerse^TM^ flow cytometer (BD Bioscience, San Jose, CA, USA).

### 4.11. Quantification of Gene Expression Using Reverse-Transcription Quantitative Polymerase Chain Reaction (PCR)

To investigate the antiproliferative and apoptotic effects of the *Streptomyces* sp. MUM256 EA, the expression of genes associated with apoptosis and cell cycle regulation were examined using qPCR. After treatment, the total RNA from cancer cells was extracted using TRIZOL reagent. The RNA concentration was determined by ultraviolet (UV) spectrophotometry. Reverse transcription of the extracted RNA was performed using a commercial cDNA reverse transcription kit. Quantitative real-time PCR was conducted in a reaction mixture containing cDNA, specific primers and SYBR Green qPCR master mix. The following primer pair sequences were used: CDK2, 5′-GGTCCTCCACCGAGACCTTAA-3′ (forward) and 5′-CAGGGACTCCAAAAGCTCTGG-3′ (reverse); CDK4, 5′-CAGTGTACAAGGCCCGTGATC-3′ (forward) and 5′-ACGAACTGTGCTGATGGGAAG-3′ (reverse); cdc25A, 5′-CCCCAAAGGAACCATTGAGA-3′ (forward) and 5′-CTGATGTTTCCCAGCAACTG-3′ (reverse); p21^WAF1/CIP1^, 5′-GGACAGCAGAGGAAGACCATGT-3′ (forward) and 5′-GCCGTTTTCGACCCTGAGA-3′ (reverse); 18S rRNA, 5′-GGCCCTGTAATTGGAATGAGTC-3′ (forward) and 5′-CCAAGATCCAACTACGAGCTT-3′ (reverse); Bax, 5′-GTCGCCCTTTTCTACTTTGCCAG-3′ (forward) and 5′-TCCAGCCCAACAGCCGCTCC-3′ (reverse); PBGD, 5′-ACCATCGGAGCCATCTGCAAG-3′ (forward) and 5′-CCCACCACACTCTTCTCTGGCA-3′ (reverse). Primers specificity was confirmed by peak melt curve before use. The amplification was then performed as described by Goh, et al. [91]. The RNA expression level of the genes of interest was then analysed and normalized to the house keeping genes (18SrRNA and PBGD) expression levels.

### 4.12. Western Blot Analysis

The cells were harvested and lysed in cold radioimmunoprecipitation assay (RIPA) buffer containing protease and phosphatase inhibitors after treatment with MUM256 EA for designated periods. The total content of protein was quantified using bicinchoninic acid (BCA) assay. Twenty-five micrograms of total protein were loaded into 10% sodium dodecyl sulphate-polyacrylamide gel electrophoresis (SDS-PAGE) and transferred onto a nitrocellulose membrane. The membrane was blocked with skim milk for 1 h prior to incubation with primary antibodies (p21, cyclin B1 and β-actin, Cell Signaling Technology, Denver, MA, USA) overnight at 4 °C. After substantial washing, the membrane was reacted with anti-rabbit/mouse immunoglobulin G-horseradish peroxidase-labelled secondary antibodies for 1 h at room temperature. The membrane was stained with enhanced chemiluminescence (ECL) detection kit prior to detection using gel documentation system. Image J was used to analyse and quantify the band intensities.

### 4.13. Hoechst 33342 Nuclear Staining

Hoechst 33342 nuclear stain was used to detect the apoptotic cells by observing the apoptotic nuclear morphology using fluorescence microscopy. After treatment, the cells were harvested in PBS and then exposed to 0.05 g/L Hoechst 33342 dye in PBS for 30 min at room temperature. The samples were observed and analysed under fluorescence microscope [90].

### 4.14. Externalization of Phosphatidylserine Detection by Flow Cytometry

The cells were harvested after treatment with MUM256 EA. A cell suspension at a density of 1 × 10^6^ /mL was prepared and subjected to centrifugation to obtain the cell pellet. The cell pellet was washed with pre-cooled PBS prior to the staining with Annexin V-FITC and propidium iodide in the 1X binding buffer [90]. After incubation at room temperature for 15 minutes in the dark, cells were analyzed by flow cytometry. A total of 10,000 events were collected and analysed using BD FACSVerse^TM^ flow cytometer (BD Bioscience, San Jose, CA, USA).

### 4.15. Measurement of Caspases-3/7 and -9 Activities

Using Carboxyfluorescein FLICA Apoptosis Detection Kit (Immunochemistry technologies, LLC), the caspases-3/7 and -9 activities were measured in HCT116 cells after treatment by flow cytometer. The treated cells were harvested and incubated with 1x FAM-DEVD-FMK or FAM-LEHD-FMK reagent for 1 h at 37 °C. The stained cells were washed twice with washing solution before subjected to 10,000 events acquisition and analysis by using FACSVerse^TM^ flow cytometer (BD Bioscience, San Jose, CA, USA).

### 4.16. Mitochondrial Membrane Potential Analysis

JC-1 (5,5′,6,6′-tetrachloro-1,1′,3,3′-tetrathylbenzimidazolcarbocyanine iodide) dye from BD^TM^ MitoScreen kit ((BD Bioscience, San Jose, CA, USA) was used to evaluate the status of mitochondrial membrane potential of the cells. After exposure to *Streptomyces* sp. MUM256 EA for a designated period, cells were harvested and washed twice before being subjected to 10,000 events acquisition and analysis by using BD FACSVerse^TM^ flow cytometer (BD Bioscience, San Jose, CA, USA).

### 4.17. Measurement of p53 and Bax Proteins by Flow Cytometry

The expression level of p53 and Bax proteins was determined by immunofluorescence staining using flow cytometry. After the treatment with MUM256 EA, cells were washed twice with PBS, fixed and subsequently permeabilized using Cytofix/Cytoperm™ kit (BD Biosciences, San Jose, CA, USA). The cells were resuspended in 500 µL of fixation/permeabilization solution and incubated at 4 °C for 20 min. The cells were washed twice with Perm/Wash™ buffer and incubated for another 15 min in 1 mL of Perm/Wash™ buffer. For the detection of Bax or p53 proteins, the fixed and permeabilized cells were incubated with 100 µL of Perm/Wash™ buffer containing corresponding antibodies. Briefly, the indirect antibodies staining was conducted by incubating the cells with mouse anti-human monoclonal antibody (p53, Bax) (Santa Cruz, TX, USA) at 4 °C in the dark for 30 min. After the washing procedure, the cells were further incubated with fluorescein isothiocyanate (FITC)-conjugated goat anti-mouse IgG_1_ (Santa Cruz, USA) at 4 °C in the dark for 30 min. Finally, the cells were then washed with Perm/Wash™ buffer before being subjected to 10,000 events acquisition and analysis by using FACSVerse^TM^ flow cytometer (BD Bioscience). FITC-conjugated normal mouse IgG_1_ (Santa Cruz, USA) was used as the isotype control to differentiate non-specific background signal.

### 4.18. Chemical Profiling of MUM256 EA Using GC-MS

Gas chromatography-mass spectrometry (GC-MS) analysis was performed to profile the constituents present in MUM256 EA [92]. The analysis was conducted using Agilent technologies 6980N (GC) equipped with 5979 Mass Selective Detector (MS), HP-5MS (5% phenyl methyl siloxane) capillary column of dimensions 30.0 m × 250 µm × 0.25 µm and helium was used as the carrier gas at 1 mL/ min. For the initial 10 min, the column was operated at 40 ºC, followed by an increase of 3 °C/min to 250 °C and was kept isothermal for 5 min. The MS was operating at 70 eV. Identification was performed by comparing the mass spectral data of the detected constituents in MUM256 EA with those available in the database NIST 05 Spectral library.

### 4.19. Statistical Analysis

All the experiments were performed at least in triplicate. The results were expressed as mean ± standard deviation (SD) and analysed using SPSS statistical analysis software. One-way analysis of variance (ANOVA) and Student′s *t*-test were performed to determine if there was a significant difference between the treated and untreated groups. A difference was considered statistically significant when *p* ≤ 0.05.

## 5. Conclusions

In summary, these findings show that MUM256 EA has an inhibitory effect on the proliferating and colony-forming abilities of HCT116 cells. Our work demonstrates that MUM256 EA induced G1 and G2/M cell-cycle arrest in HCT116 cells possibly linked to upregulation of p21 and p53 and downregulation of cyclin B1, CDK2, CDK4 and cdc25A phosphatase. Morphological changes observed suggest the occurrence of apoptosis in HCT116 cells upon treatment with MUM256 EA. The results demonstrated that MUM256 EA induced apoptosis in HCT116 cells mediated through intrinsic pathway with activation of p53, Bax, caspases-9 and -3/7. GC-MS analysis of MUM256 EA detected the presence of cyclic dipeptides which were demonstrated to have various pharmacological properties from previous literature suggesting that these compounds may have contributed to the cytotoxic properties of MUM256 EA. Furthermore, the proline-based cyclic dipeptides detected in MUM256 EA inhibited the growth HCT116 cells while showing little to no toxicity towards CCD-18Co normal colon cells. Taken together, these findings highlight the important role of exploring the microorganisms from the mangrove as a bioresource, especially for proline-based cyclic dipeptides. Further characterization of the lead compounds in this fraction may yield bioactive agents which hold tremendous promise for the development of chemopreventive drugs, and *in vivo* investigation of MUM256 EA is underway.

## Figures and Tables

**Table 1:** Chemical constituents detected by GC-MS analysis.

No.	Constituents	Retention Time	Molecular Formula	Chemical Group	Molecular Weight	Similarity (%)
1	5-Pyrrolidino-2-pyrrolidone	54.839	C_8_H_14_N_2_O	Heterocyclic, pyrrolidine	154	93.8
2	5-Isopropylidene-3,3-dimethyl-dihydrofuran-2-one	58.047	C_9_H_14_O_2_	Heterocyclic, cyclic ether	154	98.2
3	Pyrrolo[1,2-a]pyrazine-1,4-dione, hexahydro-3-(2-methylpropyl)-	58.654	C_11_H_18_N_2_O_2_	Cyclic dipeptides	210	91.6
4	4(1H)-Pyrimidinone, 6-amino-2-methyl-5-nitroso-	58.957	C_5_H_6_N_4_O_2_	Heterocyclic, pyridine	154	95.9
5	Pyrrolidine-2-carboxamide, 1-benzyloxycarbonyl-N-(4-tolyl)-	70.314	C_20_H_22_N_2_O_3_	Heterocyclic, pyrrolidine	338	96.6
6	Pyrrolo[1,2-a]pyrazine-1,4-dione, hexahydro-3-(phenylmethyl)-	71.593	C_14_H_16_N_2_O_2_	Cyclic dipeptides	244	99.0

## Supplementary Materials

The following are available online at https://www.mdpi.com/2072-6694/11/11/1742/s1: Figure S1: The Western blot of protein of interest (p21 and cyclin B) and respective β-actin expressions, Figure S2: Cytotoxic effects of the two main compounds (3) and (6) on HT-29 colon cancer cells.
