# Supplementary material for: Streptomyces sp. MUM256: A Source for Apoptosis Inducing and Cell Cycle-Arresting Bioactive Compounds against Colon Cancer Cells
Source: Cancers (Basel). 2019 Nov 6;11(11):1742. doi: 10.3390/cancers11111742 (PMC6896111; doi:10.3390/cancers11111742)
Supplement: Supplementary file 1 [file cancers-11-01742-s001.pdf]

Supplemental Materials: *Streptomyces* sp. MUM256: a source for apoptosis inducing and cell cycle arresting bioactive compounds against colon cancer cells

Loh Teng-Hern Tan, Chim-Kei Chan, Kok-Gan Chan, Priyia Pusparajah, Tahir Mehmood Khan, Hooi-Leng Ser, Learn-Han Lee and Bey-Hing Goh

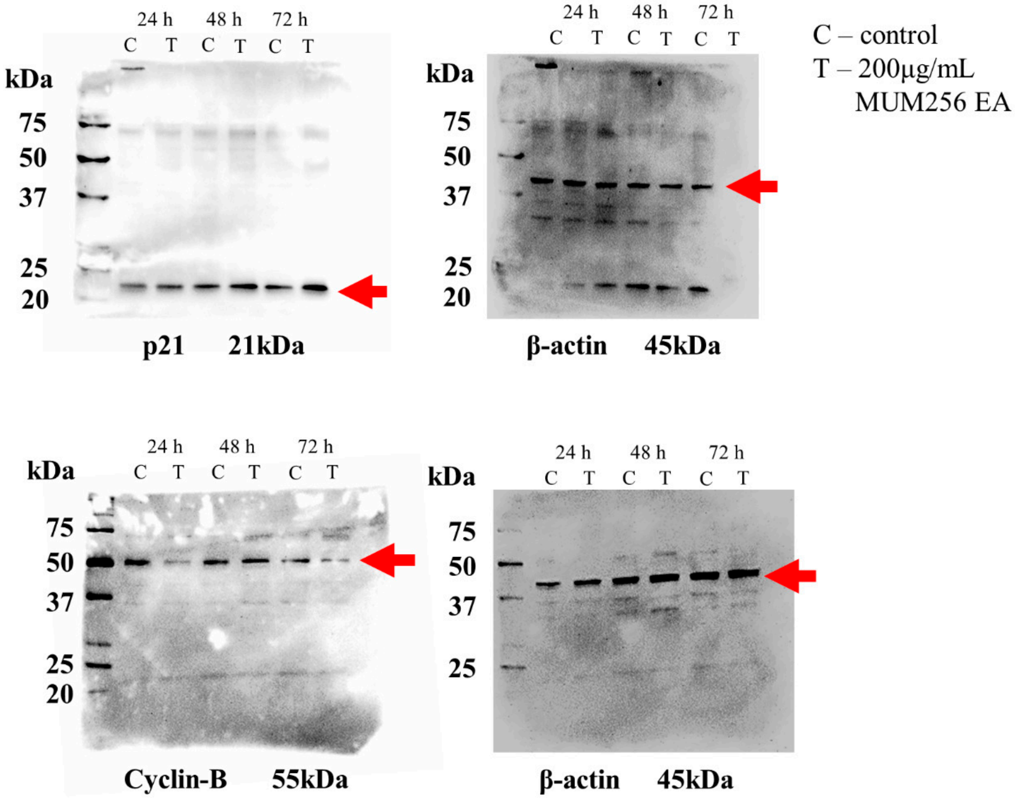

Figure S1. The Western blot of protein of interest (p21 and cyclin B) and respective β-actin expressions.

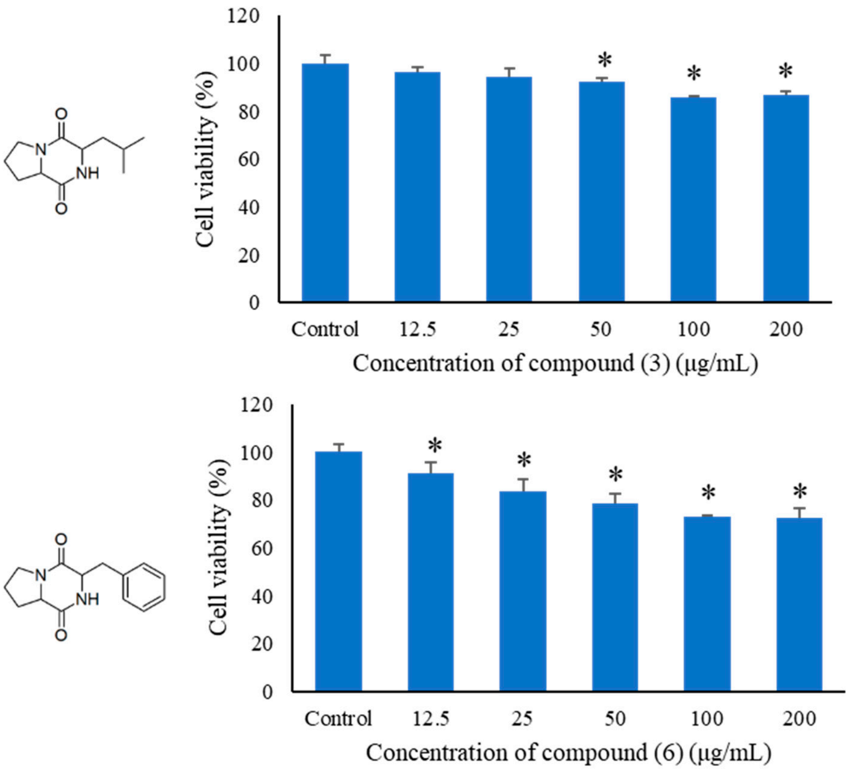

Figure 2. Cytotoxic effects of the two main compounds (3) and (6) on HT-29 colon cancer cells. Cytotoxic effect of pyrrolo[1,2-a]pyrazine-1,4-dione, hexahydro-3-(2-methylpropyl)-**(3)** and pyrrolo[1,2-a]pyrazine-1,4-dione, hexahydro-3-(phenylmethyl)-**(6)** against HT29 cells at a series of concentrations after 72 hrs treatment, ($n = 5$, * $p < 0.05$).

© 2019 by the authors. Licensee MDPI, Basel, Switzerland. This article is an open access article distributed under the terms and conditions of the Creative Commons Attribution (CC BY) license (http://creativecommons.org/licenses/by/4.0/).
